# Prevalence of antibodies to *Sarcocystis neurona* and *Neospora hughesi* in horses from Mexico

**DOI:** 10.1051/parasite/2013029

**Published:** 2013-09-10

**Authors:** Michelle R. Yeargan, Cosme Alvarado-Esquivel, Jitender P. Dubey, Daniel K. Howe

**Affiliations:** 1 Department of Veterinary Science, M.H. Gluck Equine Research Center, University of Kentucky Lexington Kentucky 40546-0099 USA; 2 Faculty of Medicine and Nutrition, Biomedical Research Laboratory, Juárez University of Durango State Avenida Universidad S/N 34000 Durango Mexico; 3 United States Department of Agriculture, Agricultural Research Service, Beltsville Agricultural Research Center, Animal Parasitic Diseases Laboratory, Building 1001 Beltsville Maryland 20705-2350 USA

**Keywords:** Seroprevalence, Equine protozoal myeloencephalitis, ELISA, Central America, Surface antigens

## Abstract

Equine protozoal myeloencephalitis (EPM) is a debilitating disease of horses caused by *Sarcocystis neurona* and *Neospora hughesi*. Sera from 495 horses in Durango State, Mexico were tested for anti-protozoal antibodies using enzyme-linked immunosorbent assays (ELISAs) based on major surface antigens of these two parasites. Antibodies to *S. neurona* were detected in 240 (48.5%) of the 495 horse sera tested with the rSnSAG2/4/3 trivalent ELISA. Multivariate analysis showed that exposure to *S. neurona* was associated with age, feeding grains and crops, and small herd size. Antibodies to *N. hughesi* were found in 15 (3.0%) of the 495 horse sera tested with the rNhSAG1 ELISA and confirmed by Western blot of *N. hughesi* tachyzoite antigen. This is the first report of *S. neurona* and *N. hughesi* exposure in horses in Mexico, and it affirms that EPM should be in the differential diagnosis for horses exhibiting signs of neurologic disease in this country.

## Abstract (fr)

La myélencéphalite équine à protozoaires (MEP) est une maladie débilitante des chevaux causée par *Sarcocystis neurona* et *Neospora hughesi*. Les sérums de 495 chevaux de l’État de Durango, Mexique, ont été testés pour les anticorps anti-protozoaires en utilisant des tests d’immuno-absorption enzymatique (ELISA) basés sur les antigènes de surface majeurs de ces deux parasites. Les anticorps contre *S. neurona* ont été détectés dans 240 (48,5 %) des sérums de chevaux testés avec ELISA contre rSnSAG2/4/3 trivalent. L’analyse multivariée a montré que l’exposition à *S. neurona* est associée avec l’âge, le nourrissage aux céréales et récoltes, et la taille des hordes. Les anticorps contre *N. hughesi* ont été trouvés dans 15 (3,0 %) des 495 sérums de chevaux testés avec ELISA contre rNhSAG1 et confirmés par western blot de l’antigène des tachyzoïtes de *N. hughesi*. Ceci est la première mention d’une exposition à *S. neurona* et *N. hughesi* chez des chevaux au Mexique, et montre que la MEP devrait être incluse dans le diagnostic différentiel des chevaux montrant des signes de maladies neurologiques dans ce pays.

## Introduction

*Sarcocystis neurona* and *Neospora hughesi* are apicomplexan protozoa that cause equine protozoal myeloencephalitis (EPM). This debilitating neurologic disease has been estimated to affect about 1 in 1000 horses annually [19] and is typically fatal if not treated. The vast majority of EPM cases are associated with *S. neurona*. Horses become infected with *S. neurona* when they ingest food and water contaminated with sporocysts or oocysts passed in the feces of the definitive host, the opossums *Didelphis virginiana* and *Didelphis albiventris* [10, 14]. Clinical disease in horses is associated with multiplication of schizonts in the central nervous system. Consistent with the geographic range of opossums, infection with *S. neurona* is limited to North, Central, and South America, with seroprevalence studies showing that horses are commonly exposed to this parasite [3–5, 7, 9, 11, 12, 16, 21–24].

The definitive host for *N. hughesi* is not known, but canids are definitive hosts for the related species *Neospora caninum.* Exposure of horses to *N. hughesi* is much lower than to *S. neurona*, but it is evident that *N. hughesi* has a wider geographic distribution since seropositive horses have been reported in the Americas, Europe, Asia, and New Zealand [2, 6–9, 11, 12, 15–17, 20, 24, 25].

The current study was conducted to assess the exposure of horses in Mexico to *S. neurona* and *N. hughesi.* The results indicated that the prevalence of antibodies to *S. neurona* is variable depending on geography but is generally high overall (approximately 50%). In contrast, antibodies to *N. hughesi* were detected in only a small proportion of the horses from Mexico, consistent with studies conducted in other parts of the world. These findings confirm that horses in Mexico are at risk of being afflicted with EPM caused by either *S. neurona* or *N. hughesi.*

## Materials and methods

Blood was collected by jugular venipuncture from 495 horses in three municipalities of Durango State, Mexico. Horse signalment and husbandry information were described previously [1]. Serum was separated by centrifugation and stored at −20 °C until used for serologic testing. The *S. neurona* trivalent recombinant protein rSnSAG2/4/3 and recombinant *N. hughesi* SAG1 (rNhSAG1) were produced and used in ELISAs essentially as described previously [17, 27]. The *S. neurona* positive control serum was from a clinically affected horse that had EPM confirmed by postmortem examination. The negative control serum was from a pre-infection foal used in a prior infection experiment [13]. The positive control sample used for the rNhSAG1 ELISA was a pool of sera from three horses that exhibited high antibody titers to *N. hughesi* (kindly provided by Dr. Nicola Pusterla, University of California, Davis, CA, USA) based on ELISA and Western blot analysis. All samples were tested in duplicate wells at a dilution of 1:250 for the rSnSAG2/4/3 ELISA and 1:500 for the rNhSAG1 ELISA. Optical density (OD) was measured at 450 nm using an *E*_max_ microplate reader (Molecular Devices). To remove interplate variation, a percent positivity (PP) relative to the controls was determined for each test sample [26]. A PP cut-off of 10% was used for the rSnSAG2/4/3 ELISA, while a cut-off of 20% was used for the rNhSAG1 ELISA; borderline PP values were rounded up to the nearest whole number (e.g., PP = 19.51 would be considered seropositive for the rNhSAG1 ELISA). At a cut-off of 20%, the rNhSAG1 ELISA was shown previously to provide 94% sensitivity and 95% specificity for detecting antibodies against *N. hughesi* [17]. The serologic accuracy of the rSnSAG2/4/3 ELISA has not yet been determined, but it is projected to provide greater than 90% sensitivity and specificity based on previous use of these SnSAG surface molecules in ELISAs [27]. To confirm results obtained with the rNhSAG1 ELISA, all samples that yielded a PP value equal to or greater than 10% were tested by Western blot analysis with *N. hughesi* whole-tachyzoite antigen, as described [17]. Samples were considered positive for antibodies against *N. hughesi* if they reacted to the two immunodominant bands that correspond to NhSAG1 and NhSRS2 [18].

Statistical analysis was performed using Epi Info software version 3.5.4 (Centers for Disease Control and Prevention: http://wwwn.cdc.gov/epiinfo/) and SPSS version 15.0 (SPSS Inc., Chicago, IL, USA). We used the Pearson’s chi-square test and the Fisher exact test (when values were less than 5) for comparison of the frequencies among groups. Multivariate analyses were used to assess the association between the characteristics of the horses and *S. neurona* and *N. hughesi* seropositivity. Variables were included in the multivariate analysis if they had a *P* value equal to or less than 0.25 in the bivariate analysis. Odd ratio (OR) and 95% confidence interval (CI) were calculated by multivariate analysis, using backward stepwise logistic regression analysis. A *P*-value of < 0.05 was considered statistically significant.

## Results

Antibodies to *S. neurona* were detected in 240 (48.5%) of 495 horses based on reactivity to the rSnSAG2/4/3 recombinant antigen (Table 1). The PP values in the seropositive samples ranged from 9.52 to 144.16, with a mean of 22.11. The seroprevalence of *S. neurona* exposure in horses varied significantly among farms (*P* < 0.001) and municipalities (*P* = 0.001) of Durango, Mexico (Table 1). Horse signalment and husbandry data and their relation with *S. neurona* and *N. hughesi* exposures are shown in Table 2. Bivariate analysis of the association of *S. neurona* seropositivity with horse characteristics showed a number of characteristics with a *P* value equal to or less than 0.25 including age (*P* < 0.004), sex (*P* = 0.07), breed (*P* = 0.007), urban area (*P* = 0.03), type of feeding (*P* = 0.01), and herd size (*P* = 0.007). Multivariate analysis of these six characteristics showed that *S. neurona* seropositivity was associated only with age (OR = 1.06; 95% CI: 1.01–1.10; *P* = 0.006), feeding with grains and crops (OR = 2.33; 95% CI: 1.17–4.66; *P* = 0.01), and small (up to 28 horses) herd size (OR = 1.94; 95% CI: 1.31–2.87; *P* = 0.0009).

**Table 1.** Seroprevalence of S*arcocystis neurona* and *Neospora hughesi* in domestic horses in Durango, Mexico.

			Seropositive to *S. neurona*		Seropositive to *N. hughesi*	
Municipality	Farm	No. of horses tested	No.	%	No.	%
Durango	DG-1	35	15	42.9	2	5.7
	DG-2	22	14	63.6	1	4.5
	DG-3	62	20	32.3	4	6.5
	DG-4	18	9	50	0	0
	DG-5	31	23	74.2	3	9.7
	DG-6	18	9	50	0	0
	DG-7	7	7	100^a^	0	0
	DG-8	25	23	92	0	0
	DG-9	3	2	66.7	1	33.3
	DG-10	28	14	50	2	7.1
	DG-11	54	30	55.6	0	0
	DG-12	42	19	45.2	1	2.4
	DG-13	27	11	40.7	0	0
	DG-14	6	4	66.7	0	0
	All	378	200	52.9^b^	14	3.7
Guadalupe Victoria	GV-1	28	6	21.4	0	0
	GV-2	19	13	68.4	0	0
	GV-3	30	5	16.7	0	0
	All	77	24	31.2	0	0
Nuevo ideal	NI-1	40	16	40	1	2.5
All		495	240	48.5	15	3

a Statistically significant difference among farms (*P <* 0.001).

b Statistically significant difference among municipalities (*P* = 0.001).

**Table 2.** General characteristics of horses and seroprevalence of *Sarcocystis neurona* and *Neospora hughesi*.

		Seropositive *S. neurona*			Seropositive *N. hughesi*		
Characteristics	No. of horses tested	No.	%	*P* value	No.	%	*P* value
Age (year)							
0.4–1	28	8	28.6	0.004	0	0	0.25
2–5	195	84	43.1		9	4.6	
6–10	176	101	57.4		2	1.1	
11–15	63	27	42.9		3	4.8	
16–22	33	20	60.6		1	3	
Sex							
Male	392	182	46.4	0.07	12	3.1	0.61
Female	103	58	56.3		3	2.9	
Breed							
Pure	385	199	51.7	0.007	10	2.6	0.22
Mixed	110	41	37.3		5	4.5	
Health status							
Ill	10	4	40	0.41	1	10	0.26
Healthy	485	236	48.7		14	2.9	
Location							
Urban	77	46	59.7	0.03	5	6.5	0.06
Rural	418	194	46.4		10	2.4	
Feeding							
Grains/crops	442	223	50.5	0.01	15	3.4	0.17
Grass	53	17	32.1		0	0	
Herd size							
3–28	201	112	55.7	0.007	4	2	0.26
30–64	294	128	43.5		11	3.7	

Antibodies to *N. hughesi* were found in 15 (3.0%) of the 495 serum samples, based on the rNhSAG1 ELISA analysis (Table 1). The ELISA PP values ranged from 20.57 to 115.68 and had a mean of 53.62. To confirm the rNhSAG1 ELISA results, Western blot analysis using *N. hughesi* whole-tachyzoite antigen was conducted on the 15 ELISA-positive sera and 33 additional sera that had ELISA PP values between 10% and 20%. This analysis revealed that 2 of the 15 ELISA-positive samples were negative for antibodies to *N. hughesi*; these sera had ELISA PP values of 21.21 and 22.13%. Two sera that had ELISA PP values between 10 and 20%, and were therefore considered negative by ELISA, tested positive by Western blot for antibodies against *N. hughesi*. One serum had an ELISA PP = 11.12 and reacted strongly in Western blot to NhSRS2 at 35 kDa but weakly with NhSAG1 at 29 kDa (data not shown). The second serum had an ELISA PP = 19.25 and recognized both surface antigens strongly in Western blot (data not shown). The remaining 31 sera with PP values between 10% and 20% were negative by Western blot for *N. hughesi* antibodies. Overall, the 15 *N. hughesi*-positive sera had a mean ELISA PP of 48.49 that ranged from 11.12% to 115.68%. Exposure to *N. hughesi* in the farms investigated varied from 0% to 33.3%. However, differences in seroprevalence among farms and municipalities were not statistically significant (Table 1).

With respect to *N. hughesi* seropositivity, characteristics with a *P* value equal to or less than 0.25 in the bivariate analysis included age (*P* = 0.25), breed (*P* = 0.22), urban area (*P* = 0.06), and feeding (*P* = 0.17). Multivariate analysis showed that none of these four characteristics were associated with *N. hughesi* seropositivity.

## Discussion

Although seroprevalence of *S. neurona* can vary widely, from 15% in wild horses in Wyoming [11] to 89% of horses in Oklahoma [3], antibodies to *S. neurona* are typically detected in 35% to 65% of horses in regions where this parasite is known to exist [4, 5, 7, 9, 12, 16, 21–24]. Therefore, the relatively high seroprevalence observed in these horses from Mexico (48.9%) is similar to what has been documented in many regions of North, Central, and South America. Interestingly, a significant proportion of Durango State is mountainous and rather arid, which has been associated with low *S. neurona* seroprevalence [11, 23, 24]. Consequently, the number of seropositive horses observed in this study was higher than might be predicted based on the geography and climate of this region. Horses were raised in the valleys region of Durango, Mexico. Exposure to *S. neurona* was associated with age, type of feeding, and herd size. The higher seroprevalence in horses fed with grains and crops than horses fed on pasture might suggest a contamination of food source in farms and a lower frequency of *S. neurona* in fields. Similarly, the association of exposure with small herd size may be related to the type of feeding. Herds of small size are commonly fed with grains and crops in stables while large herds are fed freely in the field.

As seen in multiple prior surveys [7, 9, 12, 15–17], the current study found that the proportion of horses with antibodies against *N. hughesi* was quite low (<3%). Several studies have detected antibodies to *N. hughesi* in more than 10% of horses [2, 6, 25], and even as high as 30% of horses [11, 24], and it is likely that this can be attributed partly to geographic differences. However, studies that used Western blot analysis to confirm serologic results have suggested that seroprevalence to *N. hughesi* may be commonly overestimated [7, 17, 24]. In the present study, multivariate analysis did not show an association of exposure to *N. hughesi* with any of the horse characteristics considered. However, the lack of association was potentially due to an insufficient number of positive sera to reach statistical significance.

In summary, the findings from this study show that horses in the state of Durango, Mexico are at risk of EPM. It is probable that this risk extends to other regions of Mexico, particularly where opossums are found. Without question, there are other risk factors that contribute to the development of this disease. However, the presence of the two known etiologic parasite species implies that EPM must be considered when a horse exhibits clinical signs of a neurologic disorder.
